# Misleading Robot Signals in a Classification Task Induce Cognitive Load as Measured by Theta Synchronization Between Frontal and Temporo-parietal Brain Regions

**DOI:** 10.3389/fnrgo.2022.838136

**Published:** 2022-07-01

**Authors:** Abdulaziz Abubshait, Lorenzo Parenti, Jairo Perez-Osorio, Agnieszka Wykowska

**Affiliations:** ^1^Social Cognition in Human Robot Interaction (S4HRI), Italian Institute of Technology, Genova, Italy; ^2^Department of Psychology, University of Torino, Turin, Italy

**Keywords:** cognitive control, human-robot interaction, Social Neuroergonomics, EEG, ICOH, coherence, theta, attentional orienting

## Abstract

As technological advances progress, we find ourselves in situations where we need to collaborate with artificial agents (e.g., robots, autonomous machines and virtual agents). For example, autonomous machines will be part of search and rescue missions, space exploration and decision aids during monitoring tasks (e.g., baggage-screening at the airport). Efficient communication in these scenarios would be crucial to interact fluently. While studies examined the positive and engaging effect of social signals (i.e., gaze communication) on human-robot interaction, little is known about the effects of conflicting robot signals on the human actor's cognitive load. Moreover, it is unclear from a social neuroergonomics perspective how different brain regions synchronize or communicate with one another to deal with the cognitive load induced by conflicting signals in social situations with robots. The present study asked if neural oscillations that correlate with conflict processing are observed between brain regions when participants view conflicting robot signals. Participants classified different objects based on their color after a robot (i.e., iCub), presented on a screen, simulated handing over the object to them. The robot proceeded to cue participants (with a head shift) to the correct or incorrect target location. Since prior work has shown that unexpected cues can interfere with oculomotor planning and induces conflict, we expected that conflicting robot social signals which would interfere with the execution of actions. Indeed, we found that conflicting social signals elicited neural correlates of cognitive conflict as measured by mid-brain theta oscillations. More importantly, we found higher coherence values between mid-frontal electrode locations and posterior occipital electrode locations in the theta-frequency band for incongruent vs. congruent cues, which suggests that theta-band synchronization between these two regions allows for communication between cognitive control systems and gaze-related attentional mechanisms. We also find correlations between coherence values and behavioral performance (Reaction Times), which are moderated by the congruency of the robot signal. In sum, the influence of irrelevant social signals during goal-oriented tasks can be indexed by behavioral, neural oscillation and brain connectivity patterns. These data provide insights about a new measure for cognitive load, which can also be used in predicting human interaction with autonomous machines.

## Introduction

Human operators continuously interact with machines, algorithms, avatars and robots to achieve certain goals. For example, operators rely on autonomous machines during search-and-rescue missions, space exploration and monitoring tasks (e.g., baggage screening at the airport). Since research suggests that the presence of other humans can improve operators' performance (i.e., social facilitation, reduction of errors; Krueger and Wiese, 2021), it is important to understand whether interactions with artificial agents has a similar positive effect. On face value, understanding whether interactions with artificial agents can be beneficial seems like a straightforward problem. However, due to their static nature, artificial agents may not be the best interaction partners. In this context, adaptive automated machines might be particularly helpful due to their ability to adapt their behavior to the human interaction partner (Inagaki et al., 2003; de Visser and Parasuraman, 2011; Sheridan, 2011). With adaptability of automation being a major challenge for Social Neuroergonomics (i.e., research in adaptive machines is still in ongoing; Kaber, 2018; Kohn et al., 2021; Krueger and Wiese, 2021; Calhoun, 2022), it is imperative for researchers to find implicit measures that can inform about adaptive human-robot interaction.

Studies that investigated human-robot interaction in team settings have shown positive effects of including robots in teams. For example, when people interact with robot team members, they are more empathetic toward them (de Jong et al., 2021), they perceive them as more humanlike (Fraune, 2020), as more capable of having internal states (i.e., adopting the intentional stance; Abubshait et al., 2021) and show better performance (Shah et al., 2011). More importantly, involving robots in teams can help reduce tensions that arise from failure to reach an objective (Strohkorb Sebo et al., 2018), and improve team performance overall (for a review, see: Chen and Barnes, 2014; Walliser et al., 2019). Still, human-robot teaming faces many challenges. For instance, people are reluctant to use robots, even if they were more effective in completing a task and can improve team performance (Gombolay et al., 2015). This reluctance could be due to overestimating their capabilities when working with robots, which is not evident when interacting with human partners (Gombolay et al., 2015). Even when people are willing to accept robots as teammates, issues including trust calibration, overreliance and complacency can arise (Bainbridge, 1983; Parasuraman et al., 1992; Parasuraman and Riley, 1997; Lee and See, 2004; Parasuraman and Manzey, 2010). When these issues arise, the operator is considered out-of-the-loop (i.e., OOTL; Berberian et al., 2017), which leads to detrimental problems in human-machine interaction. These detrimental issues can range in severity from simply missing an exit when driving, to the tragic loss of human lives.

To overcome these challenges of human-robot teaming/human-robot interaction, we must first identify neural indices of human-machine trust and performance (Goodyear et al., 2016, 2017; Kohn et al., 2021). Next, we are able to embed these indices in adaptive automation systems (Scerbo et al., 2003; Scerbo, 2007; Krueger and Wiese, 2021; or adaptable automation systems: Calhoun, 2022), which in turn would permit us to regulate the level of automation based on the operator's performance (Parasuraman et al., 1992; Byrne and Parasuraman, 1996; Scerbo et al., 2003; Scerbo, 2007; Feigh et al., 2012). Thus, the field of Neuroergonomics is able to overcome challenges related to trust calibration, complacency and overreliance as adaptive automation improves human performance and trust with automation (Freeman et al., 2000). More related to human-robot interaction (HRI), adaptive automation could assist researchers in understanding why robots fail to evoke brain responses in the way that robot designers intended the robots to do. For example, robots that are designed to be perceived as intentional need to engage the social brain network (Wiese et al., 2017; Perez-Osorio and Wykowska, 2020). If a robot did not engage these brain regions, it might need to adapt its behavior. To achieve adaptability, it is crucial to identify and understand neurophysiological indices of human-robot/human-automation interaction (Wang et al., 2018; Kohn et al., 2021; Choo and Nam, 2022). With this in mind, our study aimed to investigate connectivity measures in the EEG signal (i.e., synchronization measures) related to cognitive control mechanisms and attentional orienting mechanisms. This was to examine whether the interaction between these two systems can affect performance in a task that includes a virtual robot. Specifically, we employ the use of the imaginary part of Coherence (iCOH) index (Nolte et al., 2004), which measures how neural oscillations from different brain regions are related. It is imperative to capture these neurophysiological indices of cognitive control in human-robot interaction since cognitive control is a key mechanism of the brain involved in optimization of task performance. Indeed, cognitive control has been linked with reward learning and has shown the potential to influence how we interact with/on behalf of non-human interaction partners (e.g., robots, avatars, machines, and algorithms; Fedota and Parasuraman, 2010; de Visser et al., 2018; Somon et al., 2019; Abubshait et al., 2021). For example, de Visser et al. (2018) suggested that EEG components related to cognitive conflict/cognitive control are suitable neural markers to index human-machine trust and as such, they are suitable implicit indices for adaptive automated systems.

### Cognitive Control Mechanisms and Attentional Processes

Cognitive control is a mechanism that allows us to maintain and achieve goals. To do so, the brain monitors, evaluates and suppresses distracting information (Botvinick et al., 2001; Yeung et al., 2004). Experimentally, cognitive conflict/conflict monitoring has been studied using paradigms that induce conflict in the observer *via* showing task-irrelevant stimuli features (e.g., the flanker task; Eriksen and Eriksen, 1974) or by showing incongruent stimulus-response associations (e.g., Simon task; Simon and Wolf, 1963). Generally, incongruent trials are associated with longer response times and higher error rates compared to congruent trials. This difference reflects the use of more cognitive resources to resolve cognitive conflict (Botvinick et al., 2001; Yeung et al., 2004). Neurophysiological studies observed a negative oscillation 200–400 ms (i.e., N2) after the onset of conflicting information (Folstein and Petten, 2008; for a review on conflict related N2 components) with the anterior cingulate cortex (ACC) implicated as the source of this component (Botvinick et al., 2004; Yeung et al., 2004; Bocquillon et al., 2014). Conflict-related components are also present in oscillatory measurements of EEG. Studies have identified slow frequency theta oscillations (4–8 Hz) over the midfrontal cortex as an index for cognitive conflict (Sauseng et al., 2005; Moore et al., 2006; Tzur and Berger, 2007; Mitchell et al., 2008; Cavanagh et al., 2009; Yamanaka and Yamamoto, 2009; Cohen and Cavanagh, 2011; Cohen and Donner, 2013; Cavanagh and Frank, 2014; Voytek et al., 2015).

Recently, studies have shown that robot signals can also induce cognitive conflict (Perez-Osorio et al., 2021). To measure cognitive conflict, participants completed a categorization task with a virtual robot, in which they had to categorize objects based on their color, while a robot looked correctly (i.e., no conflict) or incorrectly (i.e., conflict) at one of the two target positions. Indeed, incorrect robot signals (i.e., head movements) induced cognitive conflict, which was indexed by behavioral, ocular and electrophysiological markers. This paradigm resembles the instructed saccade task (Ricciardelli et al., 2002), where participants make a choice (left or right) and wait for the go signal (gaze shift) to respond. Similarly, to the instructed saccade task studies, we found longer reaction times for incongruent gaze stimuli. Authors agree that longer reaction times reflect the interference with (oculomotor) planning and execution mainly because response selection and execution occur at different steps in the trial sequence (Dalmaso et al., 2015, 2020a,b; Porciello et al., 2016; Hietanen, 2018). Thus, the RTs reveal the cognitive conflict elicited by the social spatial cueing and the demand for cognitive control to perform the task accurately. However, the mechanisms underlying this conflict remain unknown. Specifically, whether cognitive control processes bias attentional mechanisms.

Although cognitive control mechanisms have been widely investigated, only recent studies have examined the link between cognitive control and attentional mechanisms. For instance, evidence from functional imaging (fMRI) studies revealed that when a task requires volitional attentional orienting, the brain networks involved in cognitive control communicate with attentional networks (Liu et al., 2017). The attentional networks include parietal brain regions associated in selective (Yantis et al., 2002) and sustained spatial attention (Thakral and Slotnick, 2009), and the temporal parietal junction (TPJ) linked with attention shifting (Yantis et al., 2002; Tyler et al., 2015). The link between cognitive control and attention networks has also been corroborated in studies using EEG, which postulate that theta oscillations index the communication between these two systems (Rajan et al., 2019), and that conflict-related theta can modulate attention in a spatial go/no-go task (Hong et al., 2020). Altogether, these findings suggest that decisions about where to attend are affected by the communication between cognitive-control and attention orienting systems.

### Aim of the Study

While this body of work suggests that theta power can index the communication between cognitive control and attentional systems, these studies have not directly investigated whether human performance is influenced by the modulation of connectivity between cognitive control and attentional systems. In this exploratory study, we re-examined data from Perez-Osorio et al. (2021),^1^ which evaluated cognitive conflict elicited by irrelevant robot social signals during a categorization task. In the categorization task, participants had to categorize objects based on their color. Objects were either easy or difficult to categorize. Before responding, participants viewed either congruent/correct robot head signals, in which it looked at the correct categorization location, or it incongruent/incorrect head signals where the robot looked at the incorrect categorization location. The aim was to evaluate (A) whether cognitive conflict modulates attentional systems in such collaborative scenarios and (B) how communication between cognitive control and attentional systems, as measured by functional connectivity in EEG, could predict human performance. The present work addresses these questions by first testing whether synchronization, measured by iCOH (Nolte et al., 2004), between midfrontal regions of the brain (i.e., implicated in cognitive control) and parietal regions (i.e., implicated in attention) are different when observing conflicting vs. non-conflicting robot signals. If cognitive conflict mechanisms do indeed modulate attentional system, we expected that conflicting signals of the robot recruit attentional systems more strongly and as such, we would observe a difference in coherence between congruence and incongruent conditions. To address the question of whether the effects of connectivity affect performance, we examined whether the synchronization of these two systems cognitive is correlated with reaction times of completing the task. If synchronization values and behavioral performance were indeed correlated, it would suggest that, indeed, synchronization values could assist in designing adaptive robots and machines. More formally, we hypothesized higher synchronization between cognitive-control and attentional systems for conflicting (i.e., incongruent) vs. non-conflicting (i.e., incongruent trials). Moreover, we predicted that behavioral performance could be correlated with coherence values. We did not have specific hypotheses for whether the strength of the relationship between behavioral performance and coherence values would differ based on the congruency of the head cue.

## Materials and Methods

As this paper reports results from a re-analysis of data collected for an already published paper (Perez-Osorio et al., 2021), the details of the procedures of recruiting the sample, data collection and task can be found there. Here, we report only the aspects of the study that are most relevant for the present aims. The data for this study can be found on the OSF pages of Perez-Osorio et al. (2021): https://osf.io/rpb95/ and the current study: https://osf.io/n2sw9/.

### Participants

Data used in the analyses reported in this paper are from a sample of participants recruited for the study published in Perez-Osorio et al. (2021), Experiment 3. Specifically, the sample of participants consisted of 32 participants (Median age = 23, 27 females, all right handed), which were based on an a priori analysis that was conducted with a large effect size (Cohen's dz = 0.92), a high α-error equal to 0.001 to prevent the low power typically found on EEG/ERPs studies (Clayson et al., 2019), and a power level of 0.95. The analysis yielded that a sample size of 32 participants would be sufficient to detect significant differences. Data of 2 participants were excluded due to low performance. All participants were compensated 35 euros and debriefed upon completion. The experiment was conducted in accordance with the 2013 declaration of Helsinki and was approved by a local ethical committee (Comitato Etico Rigione Liguria).

### Task and Procedure

**Figure 1:**
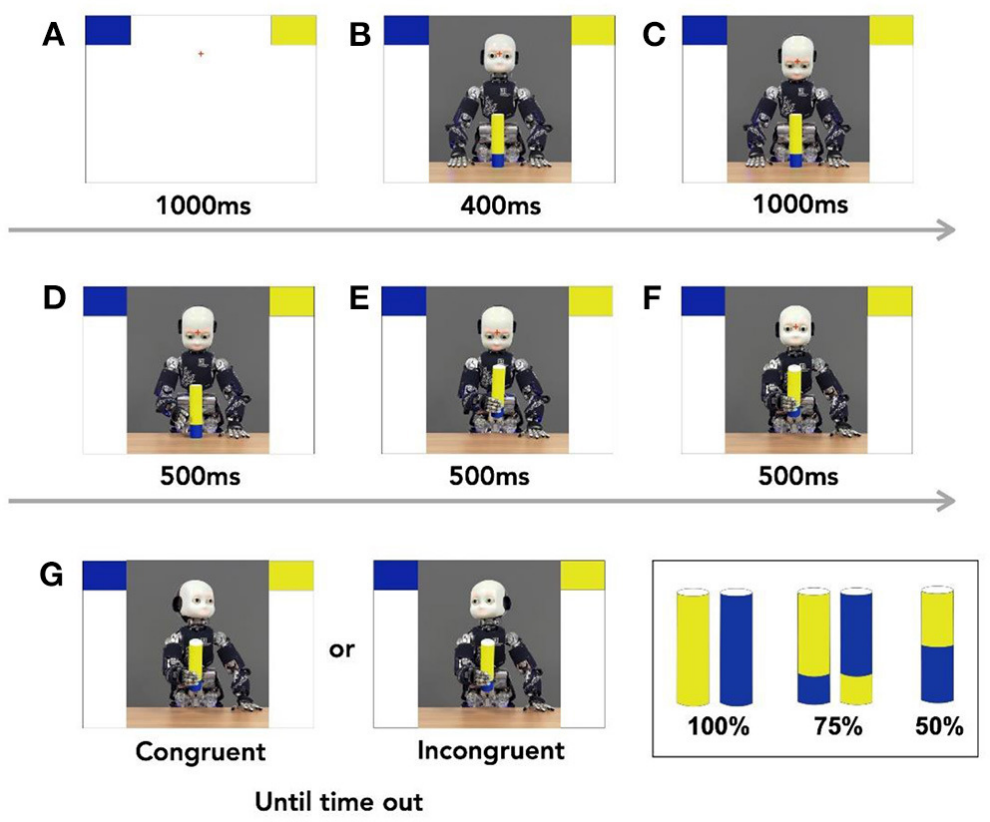
**(A–G)** Task sequence. Each trial started with two blocks appearing on the two upper corner of the screen. A picture of iCub, the humanoid robot appeared and was followed of a sequence of still images that mimicked the movement of handing over the object. After the robot completed handing over the object, it looked congruently or incongruently at one of the two bins. Participants then responded appropriately to where they thought the objects should go. The trial sequence followed the alphabetical order of the panels.

As reported in Perez-Osorio et al. (2021), participants completed an object categorization task where they categorized 5 different objects, by responding as fast and as accurately as possible. The objects included a 100% blue object, a 75% blue and 25% yellow object, a 50% blue and 50% yellow object, a 100% yellow object, and a 75% yellow and 25% blue object. On each trial, participants saw an object then decided if it belonged to one of two colored categories (i.e., blue or yellow). On each trial, a fixation cross appeared on the screen for 1,000 ms. Then, two bins appeared on the top two corners of the screen and remained on the screen until the end of the trial. One bin was blue while the other was yellow. After 1,000 ms of the onset of the bins, iCub^2^ appeared in the middle of the screen. iCub looked straight ahead with one of the 5 objects in front of it. Four hundred milliseconds later, iCub looked down at the object. Thousand milliseconds later, an image of iCub reaching for the object was shown for 500 ms. This was followed by an image of iCub grasping the object for 500 ms. Next, participants saw an image of iCub extending its arm to simulate handing over the object to the participant. Five hundred milliseconds after handing over the object, iCub looked either at the correct bin (i.e., congruent trial) or at the incorrect bin (i.e., incongruent trial). We instructed the participants to wait until iCub completed the head movement to categorize the object by pressing one of two key presses. Participants pressed “K” to choose the right bin and “D” to choose the left bin. If no response was made within 3,000 ms, the trial timed out. After categorizing the object based on color, participants saw “Correct” or “Incorrect” feedback based on their performance. The inter-trial interval (ITI) was set to 1,000 ms, see Figure 1. The position of the bins were counterbalanced across the entire experiment where the yellow bin was on the top-right and the blue bin was on the top-left 50% of the time and vice versa. The experiment also included catch trials where the robot looked down, and the participants responded by pressing the spacebar. These trials ensured that participants were paying attention to the head movements throughout the study.

Participants completed 96 trials of 100% objects, half of which were for the blue object and half for the yellow object. Participants also saw 96 trials of the 75% blue object and 96 trials for the 75% yellow object, and 96 trials for the 50% object.^3^ The robot's head movements were not predictive where it looked at the correct bin 50% of the time and at the incorrect bin 50% of the time in the correct. The experiment had 420 trials overall and took about 35 min to complete. The sequence of the trials were pseudorandomized and divided into 5 blocks with equal number of trials.

### EEG Acquisition and Preprocessing

EEG data were recorded using 64 Ag-Ag-Electrodes arranged with the 10–20 system (ActiCap, Brain Products GmbH, Munich, Germany). The data were referenced online to the Cz electrode location. Ocular movements were recorded using active electrodes located on the F9 and F10 positions for lateral movements and Fp1 and Fp2 for vertical movements. The data were recorded and amplified using a BrainAmp amplifier at a sampling rate of 500 Hz with impedances below 10 kΩ. No filters were applied online during the recording. EEG preprocessing was conducted using BrainVision Analyzer. As a first step, the EEG signal was down-sampled to 250 Hz, re-referenced to the average of the two mastoids, and then a band filter of 0.1 Hz to 30 Hz was applied. Next, we created epochs that were locked to the onset of the robot's head shift. The epochs started 1,000 ms prior to the head shift and ended 7,500 ms after the shift (i.e., epochs were 8,500 ms long). An Independent Components Analysis (ICA) was conducted on the epochs of interest to isolate and reject components related to blinks and saccades (1–2 components were rejected for each participant at most). Finally, we rejected artifacts with a maximal voltage of 20 μV/ms, a 200 μV difference in value, or a low activity of 0.5 μV for 100 ms. We rejected 6% of the trials for the Congruent/100% object, 5.6% for Congruent/75% object, 7% for the Incongruent/100% object, and 7.3% for the Incongruent/75% object. Finally, we shortened the epochs to start 1,000 ms prior to the robot's head shift to 2,000 ms after (i.e., epoch length was 3,000 ms).

To quantify how electrodes are synchronizing with one another, we used the Imaginary part of coherence (iCOH) index (Nolte et al., 2004) to determine synchronization. Coherence is a technique that quantifies the frequency and amplitude of the synchronicity of neuronal patterns of oscillating brain activity (Bowyer, 2016). This technique can be used to measure both brain synchronization between individuals (inter brain synchronization) and synchronization between brain regions of one subject (intra brain synchronization) (Sänger et al., 2012). Here, we used coherence as a measure of intra brain synchronization and we refer to intra brain synchronization as the coherence between the selected pairs of electrodes (e.g., coherence value between FCz and P1). iCOH determines the relationship between sets of electrodes. Generally, iCOH values that are closer to 0 indicate lower coherence (i.e., synchronization) and values closer to +1 or −1 indicate higher coherence. Measures of synchronization are calculated from the frequency domain representation of a pair of signals that represents an estimate of the amplitude and the phase of the oscillations across a time window (see Bastos and Schoffelen, 2016 for a comprehensive perspective). Coherence is a widely used measure to infer synchronization between different sources of signal (García Domínguez et al., 2013; Almabruk et al., 2015; Sakellariou et al., 2016). Previous studies employed iCOH on EEG data to identify differences in biomarkers of functional connectivity between children with Autism and neurotypicals (García Domínguez et al., 2013). Furthermore, intra brain differences in coherence values were also found to be predictive of performance in motor tasks (Nolte et al., 2004; Babiloni et al., 2011) and brain activity in resting-state (Kopal et al., 2014). The main issue with coherence is that it is affected by volume conduction. ICOH, proposed by Nolte et al. (2004), is developed to eliminate all sources of extraneous coherence potentially due to field spread, and captures true source interactions at a given time lag (Nolte et al., 2004, 2008).

Our motivation for using iCOH values was based on Sanchez Bornot et al.'s (2018) work that found that the original iCOH technique (Nolte et al., 2004) was one of the best methods of our functional coherence (FC) analysis. While other measurements for functional connectivity exist (e.g., PLI, wPLI), they often include additional information that empirically should improve their estimators, seem to cause loss of valuable information (Sanchez Bornot et al., 2018). According to Sanchez Bornot et al. (2018), if we have a priori information of active brain regions, and if there is a clear and non-overlapped localization for these region of interest, then FC analysis based on imaginary coherence methods, particularly iCOH, can provide useful information about the interacting neural population.

To calculate iCOH values, we first applied current source density (CSD) (orders of spline: 4, Maximal Degree of legendre polynomials: 10), CSD was obtained by applying spherical Laplace operator to the voltage distribution on the surface of the head. The procedure of spherical spline interpolation was used to calculate the total voltage distribution. The procedure followed BrainVision Analyzer recommendation for CSD procedure. The mathematical presentation of this procedure can be found in the work of Perrin et al. (1989). Next, we created pairs of electrodes between FCz (an electrode generally associated with conflict monitoring) and parietal electrodes (associated with attentional mechanisms); P1, P2, P3, P4, and Pz electrodes, specifically. We then decomposed the data by applying the Morlet complex wavelets function between 1 and 30 Hz, with 15 logarithmic frequency steps and a Morlet parameter of 5. Wavelets were normalized through Gabor normalization. To identify when theta synchronization was occurring we grand averaged coherence values of each participant selecting the frequency of interest (i.e., theta band). We performed a semiautomatic peak detection on the power spectrum relative to each of the five pairs of electrodes that we previously selected. Peak detection was set to be performed on the 600 ms after the robot cue. Finally, we selected a 100 ms time window around the peak of the theta band (i.e., 3–7 Hz) for iCOH values for all the conditions combined (time window between 252 and 352 ms, Peak at 302 ms). Data analysis was conducted on the averaged iCOH values within the 100 ms window.

## Results

Prior to running the main analyses of interest, we calculated mean and standard deviation for RTs and Coherence values. Here we report descriptive statistics for each of the two variables grouped by the type of trial: RTs: 100 congruent M = 375.74 ± 70.44; 100 incongruent M = 432.67 ± 89.28; 75 congruent M = 368.95 ± 79.29; 75 incongruent M = 431.42 ± 93.3. Coherence values: 100 congruent M = 0.036 ± 0.093; 100 incongruent M = 0.096 ± 0.12; 75 congruent M = 0.025 ± 0.091; 75 incongruent M = 0.089 ± 0.11. Next, we wanted to ensure that the coherence values that we were recording were not due to random noise therefore we performed a one-sample *t*-test to assess whether iCOH values extracted were different from zero. Results showed that Coherence values (which were averaged for each single participant) are significantly different form zero [*t*_(31)_ = 5.72, *p* < 0.001, Cohen's D = 1.01].

To test if coherence values were significantly different between congruent and incongruent conditions for each of the objects, we ran a within-factor 2 × 2 repeated measures ANOVA with congruency (congruent vs. incongruent) and object type (100 vs. 75%)^4^ as within independent variables and iCOH values as a dependent variable. To examine whether the coherence, as measured by iCOH values, between frontal electrodes and parietal electrodes can predict participant's performance (RTs), we used a linear mixed model that contained iCOH values as a continuous variable, robot head cue (Congruent = 0 vs. Incongruent = 1), as a dummy coded variable and their interaction. The mixed model also varied the intercept for each individual participant. Since the ANOVA outlined above and prior work by Perez-Osorio et al. (2021) showed that time-frequency values were not sensitive to Object-type (75% object vs. 100% object), we focused the RT analysis only on the factor congruency of the head cue.

**Figure 2:**
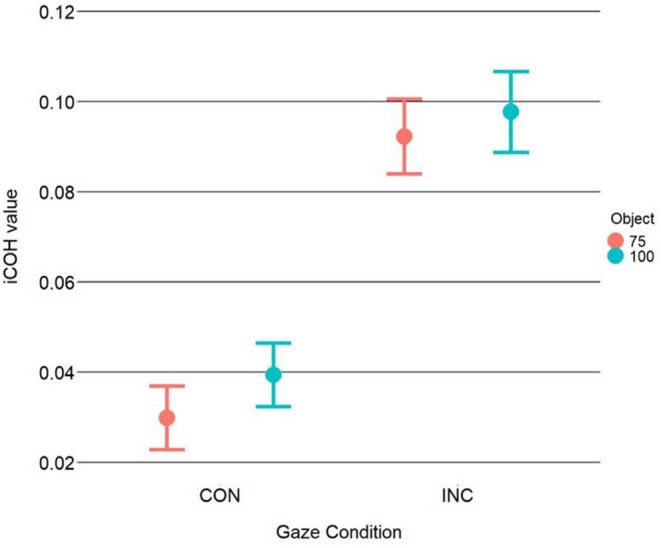
iCOH values as a function of congruency and object type. Analysis of the iCOH values shows significantly more theta synchronization between areas associated with cognitive control and those with attentional orienting for incongruent trials compared to congruent trials. This suggests that more communication is occurring in the theta range between those regions during incongruent trials. Error bars illustrate the 95% CI.

**Figure 3:**
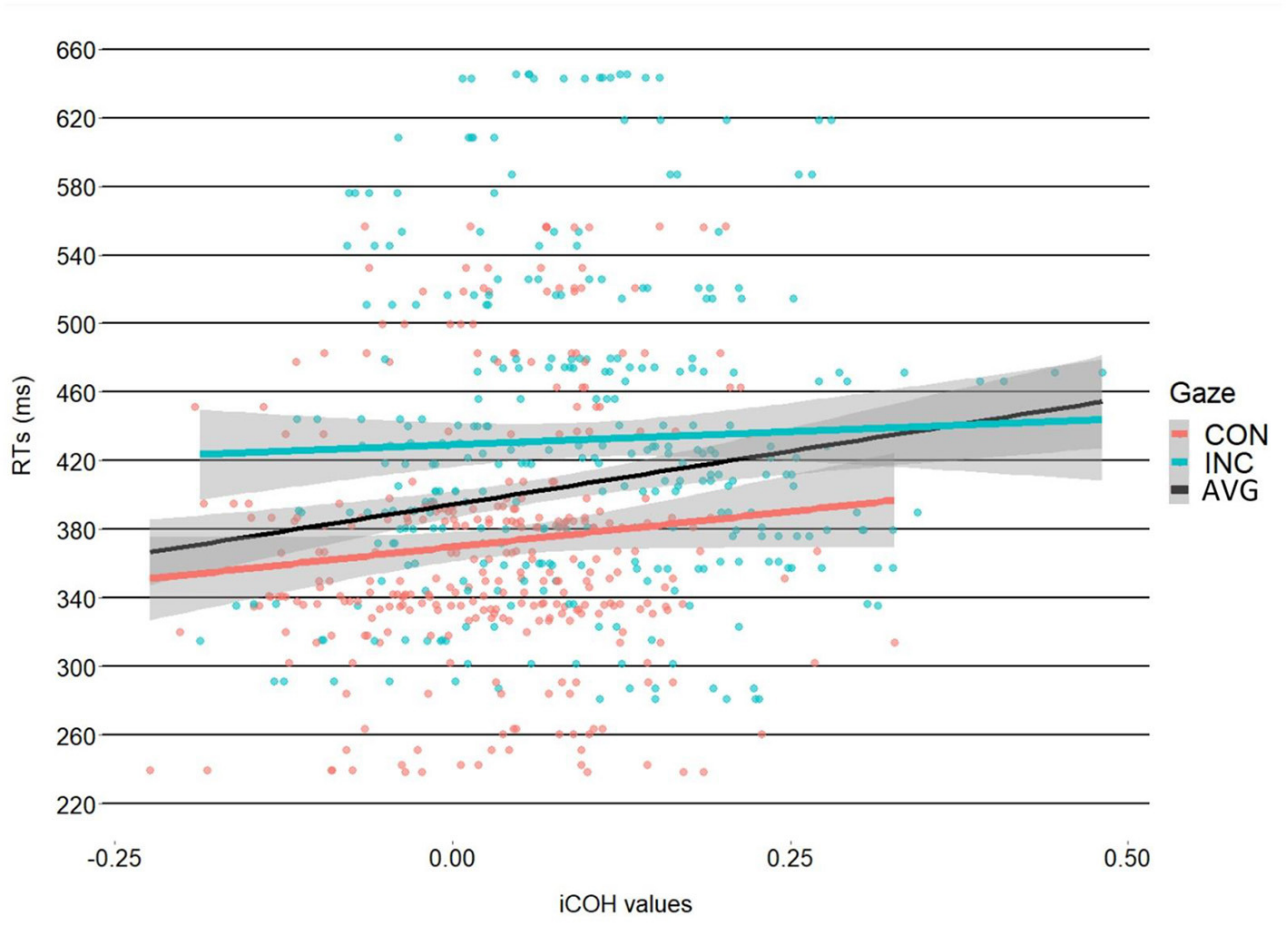
Correlation between coherence and RTs. The graph illustrates the differences in slopes between congruent (red slope) and incongruent (blue slope), with a more positive relationship between coherence and RT for congruent vs. incongruent trials. The black slope illustrates the coherence-RT relationship regardless of gaze condition. The shaded regions illustrate the 95% CI.

Results of the 2 × 2 ANOVA showed a significant effect of congruency [*F*_(1, 31)_ = 7.81, η2G = 0.2, *p* = 0.01]. The difference was such that incongruent trials had higher coherence values (M = 0.095, SD = 0.113) compared to the congruent trials (M = 0.035, SD = 0.092). The ANOVA, however, showed no effect of object-type [*F*_(1, 31)_ = 1.76, η2G = 0.05, *p* = 0.19] or a Congruency X Object type interaction [*F*_(1, 31)_ = 0.42, η2G = 0.01, *p* = 0.52], see Figure 2. After processing the data, we noticed alpha-band synchrony and as such, we ran a *post-hoc* analysis to test whether there were any differences in alpha synchronization. This seemed reasonable as prior work has shown that alpha synchrony is related to attentional resource allocation (Sauseng et al., 2005). The results showed no significant effects, however. Please see Supplementary Material for the analyses. Results of the linear-mixed model showed a significant intercept [*b* = 371, SE = 14.52, *t*_(31.25)_ = 25.54, *p* < 0.001]. The linear mixed-model showed a significant effect of the Congruency dummy variable [*b* = 60.46, SE = 2.02, *t*_(605.91)_ = 29.91, *p* < 0.001], with faster RTs for congruent trials (M = 372,3 ms, SD = 75 ms) vs. incongruent trials (M = 432 ms SD = 91.2 ms). This finding mirrors previous results reported in Perez-Osorio et al. (2021). More importantly, the analysis also showed that iCOH values significantly predicted RTs [*b* = 41.66, SE = 13.97, *t*_(605.91)_ = 2.98, *p* < 0.01] where we observed longer RTs as iCOH values increased (i.e., a positive relationship). With similar importance, we found a significant iCOH x Congruency interaction predicting participants' RTs [*b* = −36.13, SE = 17.89, *t*_(605.91)_ = −2.02, *p* = 0.04]. The interaction was such that congruent head cues were associated with a stronger relationship (i.e., more positive) between iCOH values and RTs compared to incongruent head cues, see Figure 3. This illustrates that in the congruent trials, when participants were slower to response, cognitive control systems and attentional systems were more synchronized. On the other hand, at faster RTs, these areas were more desynchronized. This effect was not evident for the incongruent trials.

One possible explanation to the unexpected finding that longer reaction times correlated with iCOH values in for the congruent trials only could be that participants experienced more load during congruent trials, which induced longer RTs and more coherence between cognitive control and attentional mechanisms. For example, participants could have experienced more load during the first half of the experiment, compared to the second half after they became more comfortable with the experiment. To verify this, we ran a *post-hoc* paired *t*-test that evaluated whether the first half of the experiment vs. the second half of the experiment in only the congruent condition were different from one another. Indeed, the *t*-test showed significant differences between the first and second half of the experiment [*t*_(33)_ = 6.75, *p* < 0.001, CI [42.11, 78.42]] with slower RTs for the first half of the experiment (M = 412 ms, SD = 73.4) compared to the second half of the experiment (M = 352, SD = 84.3). This suggest that participants experienced higher cognitive load during the first half of the experiment (longer RTs and higher coherence values) relative to the second half of the experiment.

**Figure 4:**
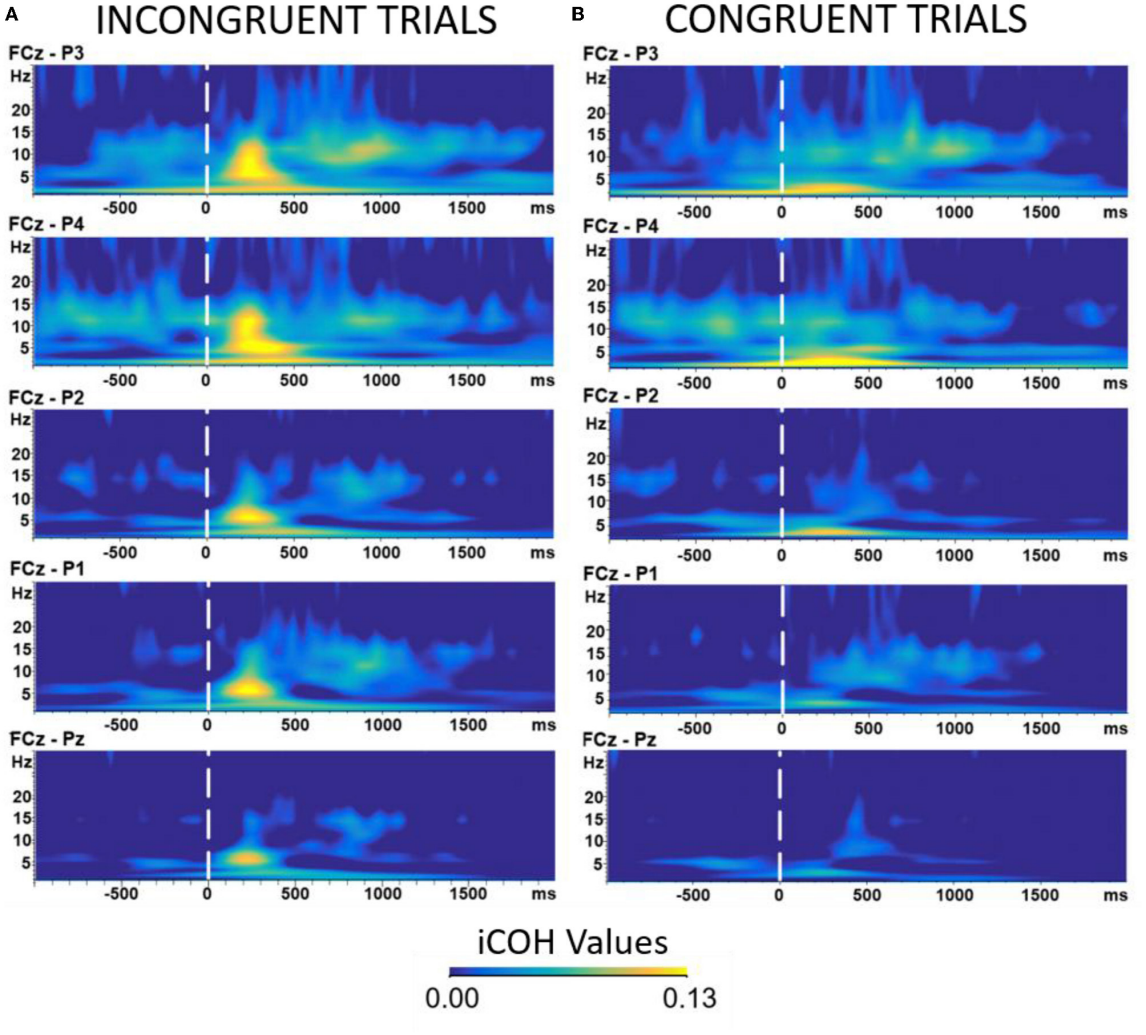
Time-frequency analysis of EEG data comparing Incongruent **(A)** vs. Congruent **(B)** trials. The epochs were all timelocked to the onset of the robot head cue. Warmer colors represent higher coherence values among the selected electrodes (see EEG acquisition and preprocessing section). The Y axis represents the frequency (measured in Hz). The X axis represents time measured in milliseconds. All results were time locked to the onset of robot's head signal. Results of the analysis showed a difference in amplitudes in the theta frequency band (3–7 Hz) at around 350–400 ms.

We additionally ran a trial-by-trial analysis on behavioral performance to test if participants' behavior changed throughout the experiment. We found that, indeed, participants were becoming faster as the experiment progressed. The details of this analysis can be found in the Supplementary Material. We also ran an analysis examining differences in iCOH values over the course of the 5 blocks of the experiment. The analysis suggested that there could be an interaction such that the iCOH values differ between objects, congruency, and over the course of the 5 blocks. However, this interaction did not show a clear pattern and could be the result of having low/different trial numbers between the conditions. See the Supplementary Material for more details and Figure 4 for the graphical illustration of the coherence analysis.

## Discussion

The aim of the current study was to investigate whether the behavioral effects of cognitive conflict that is observed in a categorization task is due to communication between cognitive control mechanisms and attentional mechanisms in the brain, as evaluated by inter-brain synchronization (iCOH). To do so, we first tested whether iCOH values were significantly different when participants observed signals that elicit cognitive conflict, compared to signals that did not elicit cognitive conflict. Specifically, we were interested in examining whether completing a categorization task with a robot that looks congruently (no/low cognitive conflict) or incongruently (cognitive-conflict) at the correct location of a target position influences the synchronization between brain areas that are related to cognitive control and attentional orienting mechanisms. We also aimed to examine whether this inter-brain synchronization is correlated with behavioral performance during a collaborative task with an autonomous agent. We tested to see if interbrain synchronization (i.e., coherence values) correlate with participants' responses (mean reaction times). Since prior work suggests that modulation of attentional orienting is due to communication between cognitive control systems and attentional systems (Liu et al., 2017), we hypothesized that completing this categorization task with the robot would influence synchronization between these two brain mechanisms. Moreover, we hypothesized that this synchronization would correlate with behavioral performance, which would lend its importance to finding a new index of successful human-robot interaction (HRI) and assist in designing adaptive autonomous systems, which is a major challenge in Social Neuroergonomics (Krueger and Wiese, 2021).

To do so, we reanalyzed an EEG dataset from an experiment in which participants completed a categorization task (see Perez-Osorio et al., 2021), where they sorted objects based on their color by selecting one of two laterally positioned bins. Before responding, participants observed iCub completing a congruent (with the target position) or incongruent (with the target position) head cue, with incongruent head cues inducing cognitive conflict (Perez-Osorio et al., 2021). We hypothesized that since incongruent trials would induce cognitive conflict, it might evoke higher degree of communication between cognitive control and attentional orienting mechanisms, as this is a postulated mechanisms allowing conflict resolution. We also hypothesized that, if iCOH is indeed a marker of cognitive control (and conflict resolution), it should relate to behavioral Results showed that coherence as measured by the iCOH index can significantly predict participants' responses. In other words, EEG indices that measure communication between mid-frontal electrodes, which are implicated in cognitive-control (Cavanagh and Frank, 2014), and parietal electrodes, which are implicated in attentional orienting (Praamstra et al., 2005; Capotosto et al., 2012), can predict how fast participants correctly classify an object. More specifically, we found that lower synchronization values (i.e., more desynchronization) correlated with slower responses, while higher synchronization correlated with longer response times. Interestingly, this effect was more evident for the congruent head cue condition than the incongruent head cue condition. This was an unexpected result but it might indicate that on slower congruent trials participants experienced more cognitive load, which could have induced variation in coherence between cognitive conflict and attentional mechanisms. This interpretation, however, should be taken with caution as it was an unexpected finding and based on a *post-hoc* analysis.

The result that incongruent trials were related with higher interbrain connectivity confirm that irrelevant social signals exhibited by an artificial agent elicit cognitive conflict. This effect is also compatible with outcome-monitoring accounts that suggest that observers had to engage cognitive control mechanisms to inhibit the interfering spatial cueing to complete the categorization task (Botvinick et al., 2004; Yeung et al., 2004). In this particular case, the cognitive conflict arises from observing an incongruent social signal that collides with the previously selected location based on the physical features of the object (i.e., the color of the object). While the response selection in congruent trials was relatively effortless, incongruent trials represented a higher cognitive demand. Concomitant information presented in incongruent trials activates two different responses when only one should be chosen, similar to classic cognitive conflict paradigms (e.g., the Stroop task; Leuthold, 2011; the Go/NoGo tasks; Nigbur et al., 2011; the Flanker task, Eriksen and Eriksen, 1974). Similar to those studies, the conflict was reflected in behavioral and neurophysiological markers (Nigbur et al., 2011; Cohen and Donner, 2013; Cavanagh and Frank, 2014). Some studies also evaluated cognitive conflict with social and non-social spatial cues (i.e., eyes vs. arrows; Marotta et al., 2018, 2019). Interestingly, incongruent eyes and arrows induce cognitive conflict (observed by behavioral markers). However, arrows elicited the opposed effect to eyes with slower responses for congruent compared to incongruent cues. Further analyses revealed that processing social stimuli requires more cognitive resources related to cognitive control and response selection (Marotta et al., 2019). In line with those findings, the current study not only shows that incongruent spatial cues induce cognitive conflict, but also provides strong evidence that the cognitive-control mechanisms are communicating with spatial attention mechanisms. This is an important new piece of information, relative to the earlier results from Perez-Osorio et al. (2021) where the authors showed how social signals from a robot can elicit cognitive conflict, but did not specify the relationship of the cognitive control mechanisms with attentional mechanisms.

Our connectivity analysis revealed how theta-band synchrony occurs between midfrontal and distal areas in a task where cognitive control is needed (Cavanagh et al., 2009; Nigbur et al., 2011). Theta-band synchrony between midfrontal and cortical areas increases after observing incongruent cues that are unexpected, which are associated to the need of control (e.g., onset of errors or conflict). This result was replicated in various studies, which highlight connectivity pattern between frontal midline electrodes and motor, sensory and prefrontal cortex (see Cavanagh and Frank, 2014 for a Review). Previous study already reported theta-band synchronization between midfrontal and parietal regions (Cohen and Van Gaal, 2013; Nurislamova et al., 2019). These results highlighted an interplay between midfrontal performance monitoring system and parietal associative areas involved in decision–making, reflecting participants behavioral adjustments and increased cognitive control in response to uncertainty (Varela et al., 2001; Nurislamova et al., 2019). Our results support the functional network proposed by Cavanagh and Frank (2014) and add to the literature about frontal mid theta as the signature of the need of control in the specific case of conflict related to social cues exhibited by an artificial agent.

The present results could also be interpreted with relation to a violation of the sense of agency. This type of violation occurs when our actions are unexpectedly overridden by an external agent or system (Padrao et al., 2016). Prior work suggests that this violation of expectation can be indexed by the N400. With respect to our results, it is possible that participants were experiencing a similar violation of sense of agency when they saw the robot looking at a bin that they did not choose, however, it is difficult to confirm whether this was the case due to the fact that it is difficult to compare data between the time domain (i.e., ERPs) and the frequency domain (i.e., ERSPs and coherence).

It is also important to note that in the current experiment, the theta-band connectivity that we observed was during viewing the robot's congruent vs. incongruent head cues as opposed to the participants committing an error. This important distinction comes from the fact that prior work has shown distinct processing of errors for when people commit errors vs. when they view others make errors (Padrao et al., 2016; Goodyear et al., 2017; de Visser et al., 2018; Weller et al., 2018; Somon et al., 2019). For instance, Weller et al. (2018) examined electrophysiological indices of error monitoring when humans commanded an agent to make an action vs. when they passively saw that agent make an action. Although they did not find differences in performance monitoring between the two conditions, they found higher Pe amplitudes for when they commanded the agent vs. when they passively saw the agent. This difference suggests that there was more attentional processing when they commanded the agent, even if it did not actually influence performance monitoring. With regards to our data, it is possible that attentional processing was hindered due to the fact that participants passively viewed the robot's head cues, but that would not necessarily affect performance monitoring. This suggests attentional processes may bias cognitive control mechanisms, but not to an extent where it would drastically affect it. This, of course, needs to be explored by future research that examines coherence between attentional and cognitive-control processing when people are monitoring performance for themselves vs. when they passively view other agents.

While we do implicate behavioral performance with communication between cognitive conflict and attentional processing mechanisms, it is not surprising that theta synchrony index this coherence. This is in light of prior work that implicates theta-band synchrony in the brain with joint/social attention, social cooperation, and interaction (Kawasaki and Yamaguchi, 2013; Dai et al., 2017; Akash et al., 2018; Wass et al., 2018; see Liu et al., 2017 for a review). While some of these studies utilized hyperscanning techniques, they still implicate inter-brain theta synchronization to these effects (Wass et al., 2018), which can shed light to the nature of the theta synchrony that we observe here.

More generally, our findings follow prior work that support the idea that implicit measures like coherence between brain regions can be used to design, implement and control agents using adaptive automation (Akash et al., 2018; Kohn et al., 2021; Krueger and Wiese, 2021; Eloy et al., 2022). For example, Akash and colleagues have shown that data from the electroencephalogram (EEG) can assist in developing human trust sensors that can implicitly predict an operator's trust levels. Similarly, Wang et al. (2018) showed that EEG data from frontal and occipital areas of the brain have the potential to predict trust. More related to our subject matter, de Visser et al. (2018) postulated that EEG activity related to cognitive conflict/cognitive control are able to serve as neural markers of human-machine interaction. In our case, the iCOH index that measures synchronization of oscillations from different brain regions (Nolte et al., 2004) has proven to be a reliable neural index of cognitive conflict. As such, this index could be used to monitor cognitive conflict from brain oscillatory behaviors. Therefore, we are able to use an iCOH index to assess whether actions or behavior from an autonomous agent exert high cognitive load on the user during collaborative tasks. For instance, depending on this index, it would be possible to select how and when to present social signals in a collaborative environment to generate fluent and efficient interaction that improves the acceptance of autonomous agents. Furthermore, such implicit indices would also help to detect when those signals produce conflict and lead to a decrease in cognitive load modifying their frequency and/or saliency.

The present work also provides additional evidence to recent work in the field of Social Neuroergonomics that illustrates the importance of understanding how the brain is responsive to social information from the external world (Jung, 2017; Dukes et al., 2021). Specifically, we show how non-informative social signals (i.e., robot head signals) can interfere with information processing. This builds on prior work that examined the directionality of information processing in the brain while completing a task with a reliable vs. unreliable automation (Goodyear et al., 2016, 2017). Both studies found that activation in the posterior insula and the left anterior precuneus was influencing subsequent activation in areas that were related to processing decision information. While our functional connectivity measure (i.e., iCOH), does not inform us about the directionality of information processing, we are able to determine if areas associated with attentional processing and cognitive conflict processing are connected based on similar signal content.

One issue to keep in mind that many of cognitive-conflict studies examined accuracy rates as behavioral data, while we used mean reaction times, as in the study of Perez-Osorio et al. (2021), participants' accuracy was very high, not allowing sufficient amount of data points for analysis. Future studies would need to design studies that focuses on error rates and not reaction times. Moreover, it would be of value to. Future work needs to also examine how connectivity between cognitive-control and attentional systems differ during tasks in which the responses of an interacting human matter (i.e., time-locking connectivity measures to responses as opposed to the onset of congruent or incongruent cues), as it could provide insight about how these two systems communicate during tasks that involve learning. Moreover, it would be of great value to the literature to understand the directionality of this flow of information. In other words, future work should use neural time series methods to understand whether oscillations from cognitive-control electrodes can causally predict oscillations in attentional orienting electrodes (e.g., granger causality analyses). Future studies should also examine the possibility of combining connectivity measures (i.e., such as iCOH) with other methods that have already been shown to improve human-machine interaction such as fNIRS (Eloy et al., 2022). For example, since work has implicated mid-frontal theta oscillations to cognitive monitoring and brain activity in the ACC (Botvinick et al., 2001; Yeung et al., 2004), this can provide us more useful information if a given task shows us differences in hemodynamic responses using fNIRS. Coherence techniques have been applied to fNIRS, MEG and EEG data successfully. Of note, the use of coherence in hyperscanning setups are more and more used in experimental psychology (for a review, see Ayrolles et al., 2021). Regardless, investigating neural indices of cognitive mechanisms involved in human-machine interaction tasks is vital for Social Neuroergonomics, as the field faces a grand-challenge of moving beyond static autonomous machines. This means that robots, machines, algorithms and avatars are currently unable (for the most part) to adapt to the human interactor's actions. As such, it is difficult to ensure successful human-machine interaction. Therefore, we suggest a neural index that could assist in designing dynamic systems that have the potential to adapt to the human user, perhaps even online.

## Data Availability Statement

The original contributions presented in the study are included in the article/Supplementary Material/OSF page: https://osf.io/n2sw9/, further inquiries can be directed to the corresponding authors.

## Ethics Statement

The studies involving human participants were reviewed and approved by Comitato Etico Rigione Liguria. The patients/participants provided their written informed consent to participate in this study.

## Author Contributions

AA, LP, JP-O and AW conceptualized the study, interpreted the data, and wrote the manuscript. AA and JP-O collected the data. AA and LP analyzed the data. All authors contributed to the article and approved the submitted version.

## Funding

This work was supported by the European Research Council (ERC) under the European Union's Horizon 2020 research and innovation program, ERC Starting Grant ERC-2016-StG-715058, awarded to AW, titled InStance: Intentional Stance for Social Attunement.

## Author Disclaimer

The content of this paper is the sole responsibility of the authors. The European Commission or its services cannot be held responsible for any use that may be made of the information it contains.

## Conflict of Interest

The authors declare that the research was conducted in the absence of any commercial or financial relationships that could be construed as a potential conflict of interest.

## Publisher's Note

All claims expressed in this article are solely those of the authors and do not necessarily represent those of their affiliated organizations, or those of the publisher, the editors and the reviewers. Any product that may be evaluated in this article, or claim that may be made by its manufacturer, is not guaranteed or endorsed by the publisher.

## Footnotes

^1^Although this data was previously published in Perez-Osorio et al. (2021), the research questions and measurements that we investigate here are entirely different and novel. Specifically, the research question was conceived and the data was obtained (i.e., EEG coherence measures) only after the previous paper was published.

^2^iCub is an open-source humanoid robot platform (Metta et al., 2010) designed to investigate the development of artificial intelligence.

^3^For the 50% object, left and right bin responses were correct. The main purpose of this condition was to evaluate whether participants would spontaneously follow the robot's gaze. They followed the gaze of the robot 75.64% of the trials in this condition (Perez-Osorio et al., 2021). Fifty percentage trials are not relevant for the current study and were not included in the data analysis.

^4^We did not examine coherence measures for the 50% object trials since participants categorized the object as they saw fit (i.e., there was no correct answer). As such, there was not a true “congruent” or “incongruent” head cue with respect to categorizing the object. Since the feedback and head cues were pseudorandomized, this could lead to spurious effects and therefore was excluded from analyses.
