# Supplementary material for: Misleading Robot Signals in a Classification Task Induce Cognitive Load as Measured by Theta Synchronization Between Frontal and Temporo-parietal Brain Regions
Source: Front Neuroergon. 2022 Jul 1;3:838136. doi: 10.3389/fnrgo.2022.838136 (PMC10790903; doi:10.3389/fnrgo.2022.838136)
Supplement: Supplementary file 1 [file Data_Sheet_1.pdf]

Supplementary materials

Analysis overtime

To examine the effect of the robot's head cues on reaction times over the course of the experiment, we conducted a linear-mixed growth curve model that tracked reaction times over the course of the experiment. The model predicted regressed time (i.e., trial number), congruency (i.e., congruent vs. incongruent), object type (i.e.,75% vs. 100%), and their interaction terms onto reaction times. The model also varied the intercept for each participant and each trial.

The analysis was done on correct trials only that was less than 1500ms and within 3SD of each condition combination for each individual. Of note, the analysis revealed a significant interaction between time and congruency (b = -.08, SE = .03, t(8874) = -2.15. p = .03), which suggests that the robot's head cues were indeed influencing participants' performance. The rest of the results can be found in table S1.

Table S1.
Results of the growth model that predicts RTs over time.

	b	*CI*	*df*	*p*
(Intercept)	430.31	398.86 – 461.75	8902	**<0.001**
Congruency	74.17	55.87 – 92.47	8902	**<0.001**
Object Type	-4.65	-20.58 – 11.27	8902	0.567
Trial number	-0.26	-0.31 – -0.21	8902	**<0.001**
Congruency X Object Type	1.60	-20.91 – 24.10	8902	0.889
Congruency X Trial number	-0.08	-0.16 – -0.01	8902	**0.031**
Object Type X Trial number	-0.01	-0.07 – 0.06	8902	0.790
Congruency X Object Type X Trial number	0.02	-0.07 – 0.11	8902	0.663

Note.

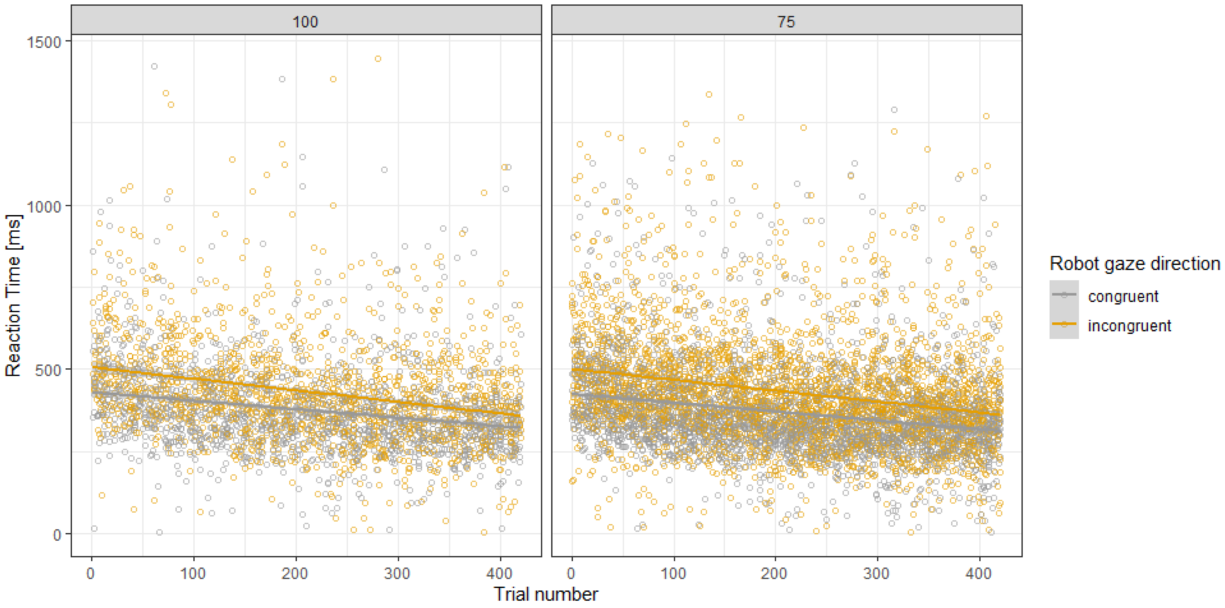

Figure S1. Behavioral performance overtime. The figure illustrates participants' behavioral results over the course of the entire experiment. The X axis illustrates the trial number, while the Y shows RTs in milliseconds. The panel on the left shows the behavioral performance for the 100% object while the panel on the right shows the 75% object. The results shows that participants were responding faster as time on task increased. The model showed that this relationship was moderated based on the congruency, but did not show an effect of object.

Coherence analysis overtime

To examine the effect of the robot's head cues on fronto-parietal synchrony over the course of the experiment, we conducted 2 x 2 x 5 within ANOVA that compared coherence between the conditions overtime. with *congruency* (i.e., congruent vs. incongruent), *object type* (i.e., 75% vs. 100%), *block number* (i.e., 1 vs. 2 vs. 3 vs. 4 vs. 5), and their interaction terms onto coherence values.

The results of the coherence analysis can be found in table S2. The main take-away is that we find significant a 3-way interaction between congruency, object type, and block number. This suggests that there be differences in congruency between those factors. To follow up on the 3-way interaction, we ran two separate 2 x 5 ANOVAs for each object type. The ANOVAs contained *congruency* and *block number* as factors. The block number interaction effect did not survive in the 75% object ANOVA. However, it did survive for the 100% object ANOVA, as shown in Table S3.

Table S2.
Results of the 2 x 2 x 5 ANOVA examining differences over the blocks.

	EtaSq	*F value*	*df*	*p*
Congruency	0.02	67.41	1, 31	**<0.001**
Object Type	0.01	34.07	1, 31	**<0.001**
Block number	0.01	4.55	4, 31	**0.001**
Congruency X Object Type	0.001	3.72	1, 31	0.053
Congruency X Block number	0.003	2.38	4, 31	0.049
Object Type X Block number	0.002	1.51	4, 31	0.196
Congruency X Object Type X Block number	0.01	4.47	4. 31	**0.001**

Note.

As such, we followed up with post-hoc t tests that compared congruent vs. incongruent conditions for each block. The post t-tests showed a significant difference in blocks 1 (p = .01), 2 (p = .003), and 4 (p < .001). However, these effects were not shown for blocks 3 (p = .33) and 5 (p = .78). Since unclear pattern of results suggests a spurious result as opposed to a learning or a fatigue effect. It could also be due to different number of trials between the blocks.

Table S3.
Results of the follow up ANOVAs.

	EtaSq	*F value*	*df*	*p*
**75% ANOVA**				
Congruency	.04	62.97	1, 31	**<0.001**
Block number	<.01	1.28	4, 31	**.27**
Congruency X Block number	<.01	1.82	4, 31	.12
**100% ANOVA**				
Congruency	.01	16.5	1, 31	**<.001**
Block number	.01	4.44	4, 31	**.001**
Congruency X Block number	.01	4.67	4, 31	**<0.001**

Note.

Alpha band analysis

We extracted coherence values from the five pairs of electrodes (FCz – P1, - P2, - P3, - P4 and - Pz) and submitted them to a two-way ANOVA. We included color of the object (75 vs 100 %) and type of gaze (congruent vs incongruent) as within-subject factors. However, there were no significant effects of the type of gaze (F(1,31) = 3.9, p = 0.06), the type of object (F(1,31) = 2.28, p = 0.14) or the gaze X object interaction (F(1,31) = 1.48, p = 0.23).
